# CD80^+^ and CD86^+^ B cells as biomarkers and possible therapeutic targets in HTLV-1 associated myelopathy/tropical spastic paraparesis and multiple sclerosis

**DOI:** 10.1186/1742-2094-11-18

**Published:** 2014-01-29

**Authors:** Soraya Maria Menezes, Daniele Decanine, David Brassat, Ricardo Khouri, Saul V Schnitman, Ramon Kruschewsky, Giovanni López, Carolina Alvarez, Michael Talledo, Eduardo Gotuzzo, Anne-Mieke Vandamme, Bernardo Galvão-Castro, Roland Liblau, Johan Van Weyenbergh

**Affiliations:** 1 Department of Microbiology and Immunology, Rega Institute for Medical Research, KU Leuven, Leuven, Belgium; 2 LIMI, Gonçalo Moniz Research Center (CPqGM), Oswaldo Cruz Foundation (FIOCRUZ), Rua Waldemar Falcão 121, 40296-710 Salvador-Bahia, Brazil; 3 INSERM UMR1043 and Pôle des Neurosciences, Hôpital Purpan, Université de Toulouse, Toulouse, France; 4 Bahiana School of Medicine and Public Health, Salvador-Bahia, Brazil; 5 Instituto de Medicina Tropical Alexander von Humboldt, Universidad Peruana Cayetano Heredia, Lima, Peru; 6 Departamento de Medicina, Facultad de Medicina Alberto Hurtado, Universidad Peruana Cayetano Heredia, Lima, Peru; 7 Instituto de Higiene e Medicina Tropical, Centro de Malária e outras Doenças Tropicais and Unidade de Microbiologia, Universidade Nova de Lisboa, Lisbon, Portugal; 8 LASP, CPqGM-FIOCRUZ, Salvador-Bahia, Brazil; 9 Institute for Immunological Investigation (iii-INCT), São Paulo, Brazil

**Keywords:** Neuroinflammatory disease, HTLV-1, Multiple sclerosis, Interferon-alpha/beta, B cell, Costimulatory CD80, CD86, Human, Ex vivo, Disease severity, Gender

## Abstract

**Background:**

Human T-cell lymphotropic virus (HTLV-1) is the causative agent of the incapacitating, neuroinflammatory disease HTLV-1-associated myelopathy/tropical spastic paraparesis (HAM/TSP). Currently, there are no disease-modifying therapies with long-term clinical benefits or validated biomarkers for clinical follow-up in HAM/TSP. Although CD80 and CD86 costimulatory molecules play prominent roles in immune regulation and reflect disease status in multiple sclerosis (MS), data in HAM/TSP are lacking.

**Methods:**

Using flow cytometry, we quantified *ex vivo* and *in vitro* expression of CD80 and CD86 in PBMCs of healthy controls, HTLV-1-infected individuals with and without HAM/TSP, and MS patients. We hypothesized *ex vivo* CD80 and CD86 expressions and their *in vitro* regulation by interferon (IFN)-α/β mirror similarities between HAM/TSP and MS and hence might reveal clinically useful biomarkers in HAM/TSP.

**Results:**

*Ex vivo* expression of CD80 and CD86 in T and B cells increased in all HTLV-1 infected individuals, but with a selective defect for B cell CD86 upregulation in HAM/TSP. Despite decreased total B cells with increasing disease duration (*p* = 0.0003, *r* = −0.72), CD80^+^ B cells positively correlated with disease severity (*p* = 0.0017, *r* = 0.69) in HAM/TSP. B cell CD80 expression was higher in women with HAM/TSP, underscoring that immune markers can reflect the female predominance observed in most autoimmune diseases. In contrast to MS patients, CD80^+^ (*p* = 0.0001) and CD86^+^ (*p* = 0.0054) lymphocytes expanded upon *in vitro* culture in HAM/TSP patients. The expansion of CD80^+^ and CD86^+^ T cells but not B cells was associated with increased proliferation in HTLV-1 infection. *In vitro* treatment with IFN-β but not IFN-α resulted in a pronounced increase of B cell CD86 expression in healthy controls, as well as in patients with neuroinflammatory disease (HAM/TSP and MS), similar to *in vivo* treatment in MS.

**Conclusions:**

We propose two novel biomarkers, *ex vivo* CD80^+^ B cells positively correlating to disease severity and CD86^+^ B cells preferentially induced by IFN-β, which restores defective upregulation in HAM/TSP. This study suggests a role for B cells in HAM/TSP pathogenesis and opens avenues to B cell targeting (with proven clinical benefit in MS) in HAM/TSP but also CD80-directed immunotherapy, unprecedented in both HAM/TSP and MS.

## Background

Human T-cell lymphotropic virus 1 (HTLV-1) was the first human retrovirus to be isolated [1]. In contrast to HIV, the provirus preferentially replicates through oligoclonal proliferation of infected cells rather than by new virion production [2]. Although estimates suggest that about 10–20 million people worldwide are HTLV-1 seropositive [3], a recent review by Hlela et al. suggests that this number could be underestimated [4]. While most of the HTLV-1 seropositive individuals remain asymptomatic lifelong, a minority of HTLV-1-infected individuals progress to severe, often fatal disease [5]. The more predominant complications are adult T-cell leukemia/lymphoma (ATL) and HTLV-1-associated myelopathy/tropical spastic paraparesis (HAM/TSP) [5]. This disparity in disease outcome is still not well understood, but is probably determined by the interaction between viral, immune and host genetic factors [6].

HAM/TSP is a chronic progressive inflammatory disorder causing degenerative myelopathy. It evolves progressively from inception, with spastic paraparesis or paraplegia being the principal clinical condition, accompanied by bladder dysfunction and sensory deficit in the lower extremities [5]. The impact of disease severity is presented by two commonly used scales: Kurtzke’s Expanded Disability Status Score (EDSS) [7] and the Osame Motor Disability Score (OMDS) [8]. Given that the host’s immune system is generally considered responsible for inflicting inflammatory damage to the spinal cord [9], various therapeutic approaches, mostly immunomodulators and antivirals, have been adopted in HAM/TSP. Corticosteroids such as prednisolone and hydrocortisone as well as the immunomodulatory cytokine interferon (IFN)-α have shown some clinical benefit [10]. However, effective or disease-modifying therapy is still unavailable [11], as are bona fide biomarkers for disease progression and/or therapeutic failure. Proviral load is currently the most widely used biomarker in HAM/TSP research, although it did not reflect therapeutic response in recent clinical trials using antiretrovirals, valproic acid, or IFN-α or IFN-β [12-15].

An increased T-cell activation, uncontrolled lymphocyte proliferation [16] and proinflammatory cytokine production in HTLV-1-infected subjects have been associated with the development of disease [17]. *Ex vivo* findings include high proviral load in peripheral blood mononuclear cells (PBMCs) [18] and proinflammatory cytokines such as tumor necrosis factor (TNF)-α, interleukin (IL)-6 and IFN-γ in the serum and cerebrospinal fluid (CSF) [19-21]. Neuropathological analysis revealed T cell (CD4^+^ and CD8^+^) dominant, mononuclear cell infiltration [9]. In addition to preferential infection of T cells, the virus is also known to infect antigen-presenting cells (APCs), namely dendritic cells, B cells and macrophages, which regulate T cell fate in vivo [22,23]. An inflammatory process depends on T cell activation, which requires engagement of the T cell receptor (TCR) with the MHC-peptidecomplex presented on the cell surface of APCs. In addition to this antigen-specific stimulation, a second interaction involving a costimulatory molecule, CD28, on T cells and its ligands, CD80 (B7.1) and CD86 (B7.2), on APCs is required for optimal T cell activation [24]. Further, these two signals do not need to be delivered concomitantly for optimum T cell activation [25]. In HAM/TSP patients, costimulatory molecules on APCs induced by viral tax provide constant antigen presentation and costimulation to T cells, leading to intense T cell proliferation and inflammatory responses [26]. Interestingly, expression of CD80 and CD86 is not restricted to APCs, but may be expressed in T cells of HTLV-1-infected individuals [27]. The use of anti-CD80 and anti-CD86 antibodies inhibited spontaneous proliferation of lymphocytes. In addition, simultaneous addition of anti-CD80 and anti-CD86 antibodies inhibited production of IFN-γ, TNF-α and IL-4, with no effect on IL-10 production for both, allo- and autologous T cell proliferation. Taken together, these results suggest that HTLV-infected CD80^+^/CD86^+^ T cells could also serve as APCs, enabling a sustained proliferation of T cells [26].

In EAE, a mouse model for MS, the blocking of the costimulatory molecules CD80 and CD86 in peripheral blood cells and the use of CD80/CD86 knockout mice provide evidence of their pathogenic role [28-30]. Interestingly, even reactive astrocytes may potentially share the functions of APCs given their expression of CD80 and CD86 [31]. While data are lacking on the expression of CD80 and CD86 in HTLV-1 infection and pathogenesis, IFN-α enhanced CD80 expression *in vitro* in myeloid leukemia [32], while IFN-β has been shown to regulate CD80 and CD86 *in vivo* and *in vitro* in MS [33,34]. IFN-β treatment also reduced CD80-induced IL-2 producing cells *in vitro*[35]. Taken as a whole, modulation of CD80 and CD86 costimulatory molecules occurs in different cell types and is postulated to participate in MS pathogenesis.

In addition, in MS, IFN-β, one of the current first-line therapies [36], has been effective in numerous therapeutic trials and has been widely used in the last 2 decades [37,38]. In HAM/TSP, both IFNs have been tested in only a few therapeutic trials [10,14,15,39,40], while their mechanism of action remains enigmatic. We hypothesized that *ex vivo* expression of CD80 and CD86 as well as the *in vitro* effects of IFN-α and IFN-β on their expression could reveal biomarkers for possible clinical use in HAM/TSP.

## Patients and methods

### Sampling

This study was approved by the Ethics Committee of the Oswaldo Cruz Foundation (FIOCRUZ), Salvador-Bahia, Brazil, Universidad Peruana Cayetano Heredia, Lima, Peru, and Hôpital La Pitié-Salpêtrière, Paris, France. A total of 55 individuals, including 23 healthy controls (HCs), 6 HTLV-1-infected individuals without HAM/TSP (asymptomatic carriers, ACs) and 26 HAM/TSP patients (9 men and 17 women) were recruited from two endemic regions (Salvador-Bahia, NortheEast Brazil, and Lima, Peru) following written informed consent. HAM/TSP was diagnosed by the Osame criteria (based on WHO guidelines) [41]. Antibodies to HTLV-I/II were investigated by diagnostic enzyme-linked immunosorbent assay (ELISA, Cambridge Biotech, Worcester, MA, USA) and confirmed by Western blot capable of discriminating between HTLV-I and HTLV-II (HTLV Blot 2.4, Genelab, Singapore; Abott Diagnostics, USA; Murex Diagnostics, UK, or Biokit, Spain). Proviral load (which is the viral DNA integrated in the host cellular genome) in HAM/TSP patients and ACs was quantified according to Grassi et al. in Brazil [42] and Adaui et al. in Peru [43]. In the MS cohort, 20 patients with relapsing/remitting MS, 5of whom had stable disease, analyzed at baseline and 1 month after *in vivo* treatment with IFN-β1a (30 μg administered intramuscularly, once weekly), were recruited at Hôpital La Pitié-Salpêtrière, Paris, France, following provision of written informed consent.

### Cell culture

PBMCs were obtained from 5-10 ml of heparinized venous blood by passage over a Ficoll Hypaque gradient (Sigma-Aldrich). PBMCs were washed twice with PBS and resuspended at a concentration of 4 × 10^6^ cells/ml in RPMI1640 medium supplemented with 2 mM L-glutamine, gentamycin (50 μg/ml) and 10% heat-inactivated fetal calf serum (all fromLife Technologies, NY). Cells were plated in 24-well tissue culture plates (Costar, Corning Incorporated, NY) at a concentration of 4 × 10^6^ cells/ml and incubated at 37°C and 5% CO_2_. *In vitro* stimulation was performed by addition of IFN-α2a (1,000 U/ml) or IFN-β1b (1,000 U/ml), or anti-CD3 as a positive control. Cells were collected at 48 h and stained for flow cytometry.

### Flow cytometry

**Figure 1:**
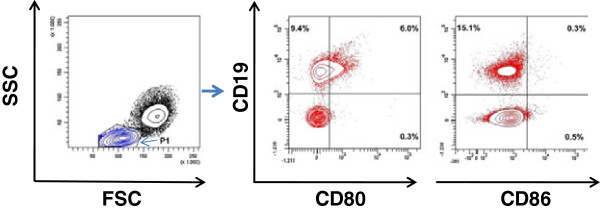
**Gating strategy for CD80 and CD86 quantification in PBMCs.** PBMCs from a representative HAM/TSP patient were stained with CD3-, CD19-, CD80- and CD86-specific monoclonal antibodies and analyzed by flow cytometry. P1 (in *blue contours*) represents the lymphocyte gate. Percentages of CD80^+^ and CD86^+^ cells within the lymphocyte gate (P1) are indicated in the respective quadrants (*red contours*).

For phenotypic analyses, PBMCs were resuspended at a density of 200,000 cells in 50 μl of 1% BSA plus 0.1% sodium azide in PBS and incubated for 30 min on ice with mAbs specific for CD3, CD4, CD8, CD19, CD80, CD86 and the corresponding isotype controls (BD Biosciences). A minimum of 10,000 events per sample was acquired with FACSort and FACS CantoII flow cytometers (BD Biosciences) and analyzed using CellQuest and Diva software, respectively. Figure 1 shows dot plots of a representative HAM/TSP patient to indicate the gating strategy for CD80 and CD86 quantification in B and T cells. For proliferation analysis, after 48 h of *in vitro* culture, PBMCs were stained with the antibody cocktail (listed above) and Hoechst 33342 and incubated for 15 min in the dark at room temperature before acquisition. Proliferating (tetraploid, 4n) lymphocytes were quantified in specific (CD80^+^ and CD86^+^ T and B cell) gates based on MFI of Hoechst 33342, which is twice that of the diploid (2n) gate, representing lymphocytes with normal DNA content. Doublets (gated using width vs. area dot plots) and sub-diploid/apoptotic cells (gated using DNA content vs. side scatter) were excluded.

### Statistical analysis

The use of parametric versus non-parametric tests was decided based upon the Kolmogorov-Smirnov test for normality. These included paired and unpaired *t* test, repeated measures ANOVA with Bonferroni’s multiple comparison test or Kruskal-Wallis test with Dunn’s post test, Spearman’s correlation analysis and receiver-operator characteristic (ROC) curve analysis. All tests were two-tailed, and differences were considered significant at *p* values <0.05.

## Results

### Clinical and demographic data

All HTLV-1 infected individuals were serologically positive for HTLV-1 and negative for HTLV-2. The median proviral load was 2,782 copies/10^4^ PBMCs (range: 6.3–3,805 copies/10^4^ PBMCs).

The mean age of HAM/TSP patients was 49.5 ± 2.1 years (range: 27–64 years) and mean duration of disease was 7.0 ± 1 years (range: 0.8–20 years). EDSS ranged from 3–7 (mean 4.8 ± 0.3). For a subgroup of Brazilian patients, the Osame Motor Disability Scale was also available. We observed a significant positive correlation between Kurtzke’s EDSS score and the Osame score (*p* = 0.001, *r* = 0.81, *n* = 12), similar to the findings of Olindo et al. in a group of 100 patients from the French WestIndies [44]. The mean age of HCs was 33.9 ± 3.8 years (range 23.6–43.8 years), while that of ACs was 40 ± 7.8 years (range 9–64 years).

### Increased *ex vivo* frequency of CD80^+^ and CD86^+^ lymphocytes in HTLV-1-infected individuals and selective loss of B cell CD86 upregulation in HAM/TSP patients

**Figure 2:**
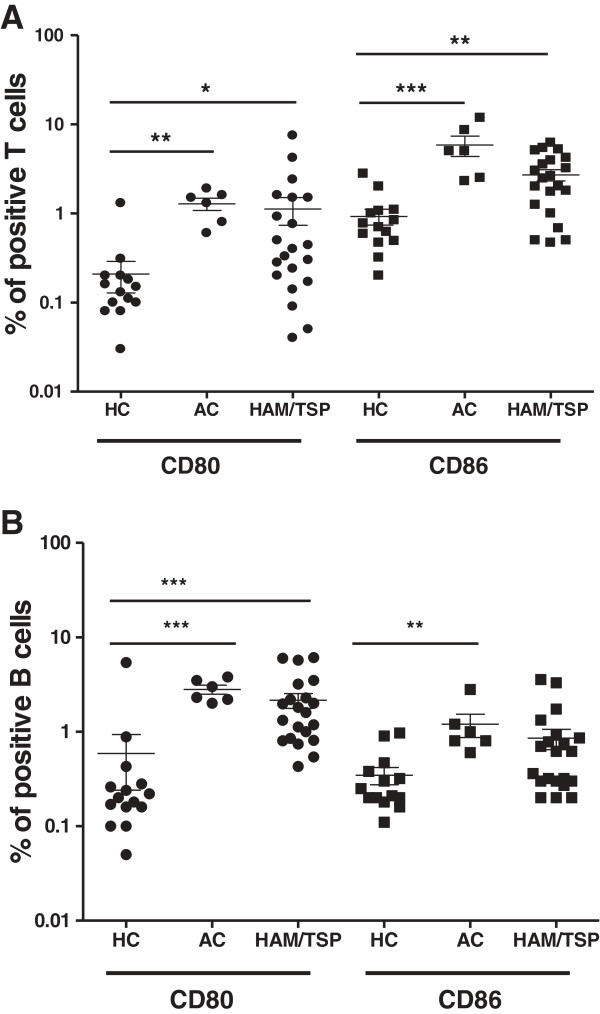
**Increased *ex vivo *expression of CD80 and CD86 in T and B cells of HAM/TSP patients and HTLV-1 asymptomatic carriers (ACs).** CD80 and CD86 expression in (A) T (CD3+) cells and (B) B (CD19+) cells of HCs (n = 15), ACs (n = 6) and HAM/TSP patients (n = 21) were quantified using flow cytometry. (*p < 0.05, **p < 0.01, ***p < 0.001; Kruskal-Wallis test with Dunn’s multiple comparison post test).

We quantified the surface expression of CD80 and CD86 in T and B lymphocytes in HCs (*n* = 15), ACs (*n* = 6) and HAM/TSP patients (*n* = 21) by flow cytometry. As shown in Figure 2, we observed a significant increase in the percentage of *ex vivo* CD80^+^ T cells (CD3^+^CD19^-^) as well as CD86^+^ T cells between infected and uninfected subjects. Furthermore, CD80^+^ B cells (CD3^-^CD19^+^) were significantly enhanced in HTLV-1-infected individuals when compared to HCs. Interestingly, there was a significant increase in CD86^+^ B cells only in ACs but not in HAM/TSP patients when compared to HCs, indicating a selective loss in HTLV-1 upregulation of B cell CD86 upon disease progression to HAM/TSP. However, expression of CD80 and CD86 on a per-cell basis, as mean fluorescence intensity (MFI), did not differ significantly among HCs, ACs and patients in either cell type (data not shown).

In addition, in HAM/TSP patients, we observed a positive correlation between the *ex vivo* expression of CD80 and CD86 in T cells (*p* = 0.039, *r* = 0.50, *n* = 18) but not in B cells (*p* > 0.05,*n* = 17) (data not shown). This implies that CD80 and CD86 expression may be differentially regulated in HAM/TSP only, since there was no significant correlation in *ex vivo* expression of CD80 and CD86 in any cell type in HCs and ACs (data not shown).

### CD80^+^ B cell expression is positively correlated to disease severity and is gender biased in HAM/TSP patients

**Figure 3:**
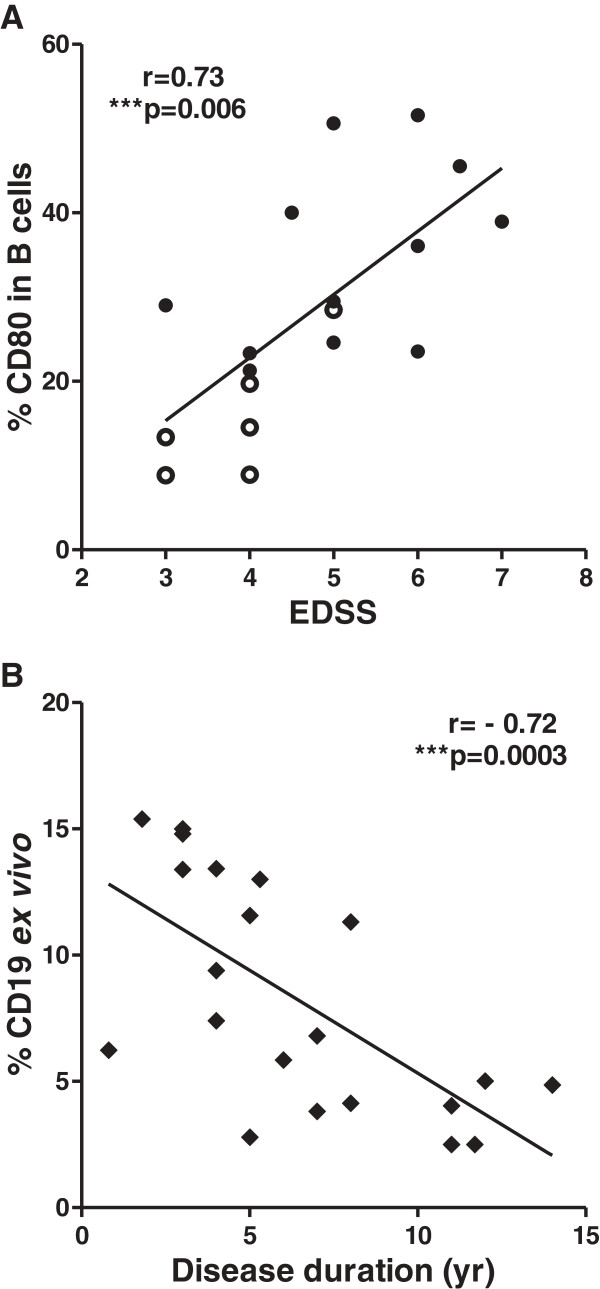
***Ex vivo *levels of CD80**^**+ **^**B cells correlate with disease severity, whereas total B cell (CD19**^**+**^**) levels decrease overtime in HAM/TSP patients. (A)** Positive correlation between the percentage of CD80 in B cells (CD19^+^) and Kurtzke’s EDSS. (***p = 0.0006, Spearman’s *r* = 0.73, *n* = 18). **(B)***Ex vivo* CD19^+^ B cell level is negatively correlated to disease duration in HAM/TSP patients (*n* = 21). (***p = 0.0003, Spearman’s *r* = 0.72, *n* = 21).

**Figure 4:**
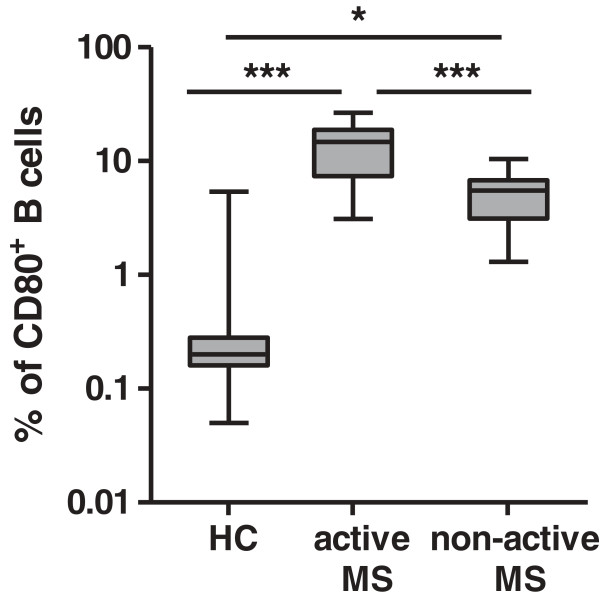
**Increased *ex vivo *expression of CD80 in B cells of MS patients.** MS patients with active disease (*n* = 6) expressed significantly higher levels of CD19^+^CD80^+^ than MS patients with non-active disease (*n* = 10), and both groups were higher in CD19^+^CD80^+^ expression than HCs (*n* = 15). (*p < 0.05, ***p < 0.001; ANOVA with Bonferroni’s multiple comparison post test).

To explore the possible clinical relevance of increased CD80 and CD86 on B and T cells, we correlated flow cytometry results to clinical and demographic patient data. We observed that the percentage (but not MFI) of CD80-expressing B cells but not CD80-expressing T cells or total lymphocytes positively correlated with disease severity as measured by Kurtzke’s EDSS (*r* = 0.73, *n* = 18) (Figure 3A). Expression of CD86 (either as % or as MFI) in B or T cells as well as total lymphocytes did not correlate to disease severity (data not shown). Interestingly, the ratio of CD86:CD80 expression in B cells but not in T cells correlated negatively to disease severity in HAM/TSP patients (*r* = −0.50, *n* = 18) (Additional file 1: Figure S1), implying a possible antagonism between the two costimulatory molecules, as previously suggested by Genç et al. in MS patients [33]. Further, the percentage of total CD19^+^ B cells negatively correlated to disease duration (*r* = −0.72) (Figure 3B) but not to age or EDSS. Notably, proviral load, disease duration and age did not correlate to disease severity (data not shown). Therefore, the increase in CD80 expression in B cells in HAM/TSP patients, which occurs despite a decrease in total B cell levels, likely reflects disease progression rather than prolonged infection or disease duration. Our findings in HAM/TSP patients are highly similar to those in MS patients. We observed a significant increase in CD80 expression in B cells of MS patients with both active and non-active disease as compared to HCs (Figure 4), in agreement with Genç et al. [33]. Given the decrease in total B cell levels in HAM/TSP patients (Figure 3B), we used relative CD80 B cell expression (CD80^+^:CD19^+^ ratio) in HAM/TSP and absolute values of CD80-expressing B cells (CD19^+^CD80^+^) in MS. Using receiver-operating characteristic (ROC) curves, we found that the CD80^+^:CD19^+^ ratio and CD19^+^CD80^+^ levels could discriminate between patients with impaired mobility in HAM/TSP (*p* = 0.0010, AUC = 0.96) and patients with active MS (*p* = 0.0077, AUC = 0.88), respectively. The best performing cutoffs were 0.23 for the CD80^+^:CD19^+^ ratio and 8.4% for CD19^+^CD80^+^, respectively (Additional file 2: Figure S2 A, B).

**Figure 5:**
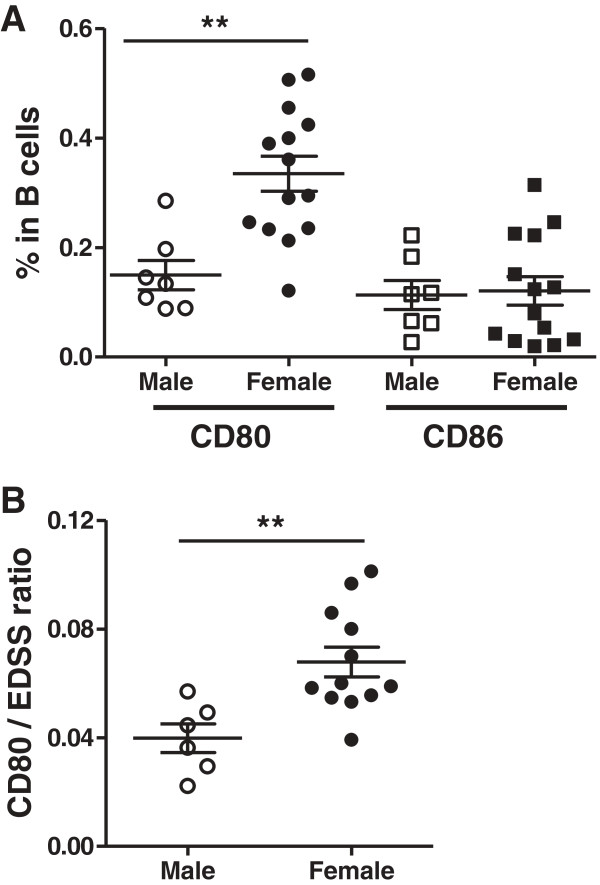
**Gender-biased expression of CD80 but not CD86 in B cells of HAM/TSP patients. (A)** Expression of CD80 but not CD86 in B cells is increased in female (*n* = 14) versus male (*n* = 7) patients (**p = 0.0013, unpaired *t* test). **(B)** B cell CD80 expression corrected for disease severity (EDSS) is significantly higher in female (*n* = 12) versus male (*n* = 6) HAM/TSP patients. (**p = 0.0054, unpaired *t* test).

In addition, the percentage of CD80^+^ B cells but not CD80^+^ T cells or CD80^+^ total lymphocytes was significantly higher (2.3 fold) in female (*n* = 14) versus male (*n* = 7) patients (Figure 5A), whereas CD86 expression was not significantly different between female and male patients in any lymphocyte population (Figure 5A and data not shown). There is a predominance of HAM/TSP in women [45], who were also found to have more severe disease and to progress faster [46]. However, the association of CD80^+^ B cells with both female gender and disease severity was not interdependent. As shown in Figure 5B, CD80^+^ B cell levels corrected for EDSS were still significantly higher in female vs. male HAM/TSP patients (*p* = 0.0054, unpaired *t* test). It would be interesting to confirm these findings in HAM/TSP patients of other endemic regions as well as in large MS cohorts.

### CD80^+^ and CD86^+^ lymphocytes expand upon *in vitro* culture in HAM/TSP but not MS patients

The decrease in *ex vivo* total CD19^+^ B cells in HAM/TSP patients over time (Figure 3B) somehow contrasts with the observed *ex vivo* increase in CD80^+^ B cells. Hence, increased CD80 levels in B cells could be due to proliferation of a specific B cell subset in HAM/TSP. Low but detectable proliferation of total B cells has been demonstrated *in vitro* for HAM/TSP patients [47,48], albeit significantly lower than that of CD8, CD4 or NK cells [47]. Therefore, we examined the effect of *in vitro* culture upon CD80 and CD86 expression as compared to *ex vivo* levels.

**Figure 6:**
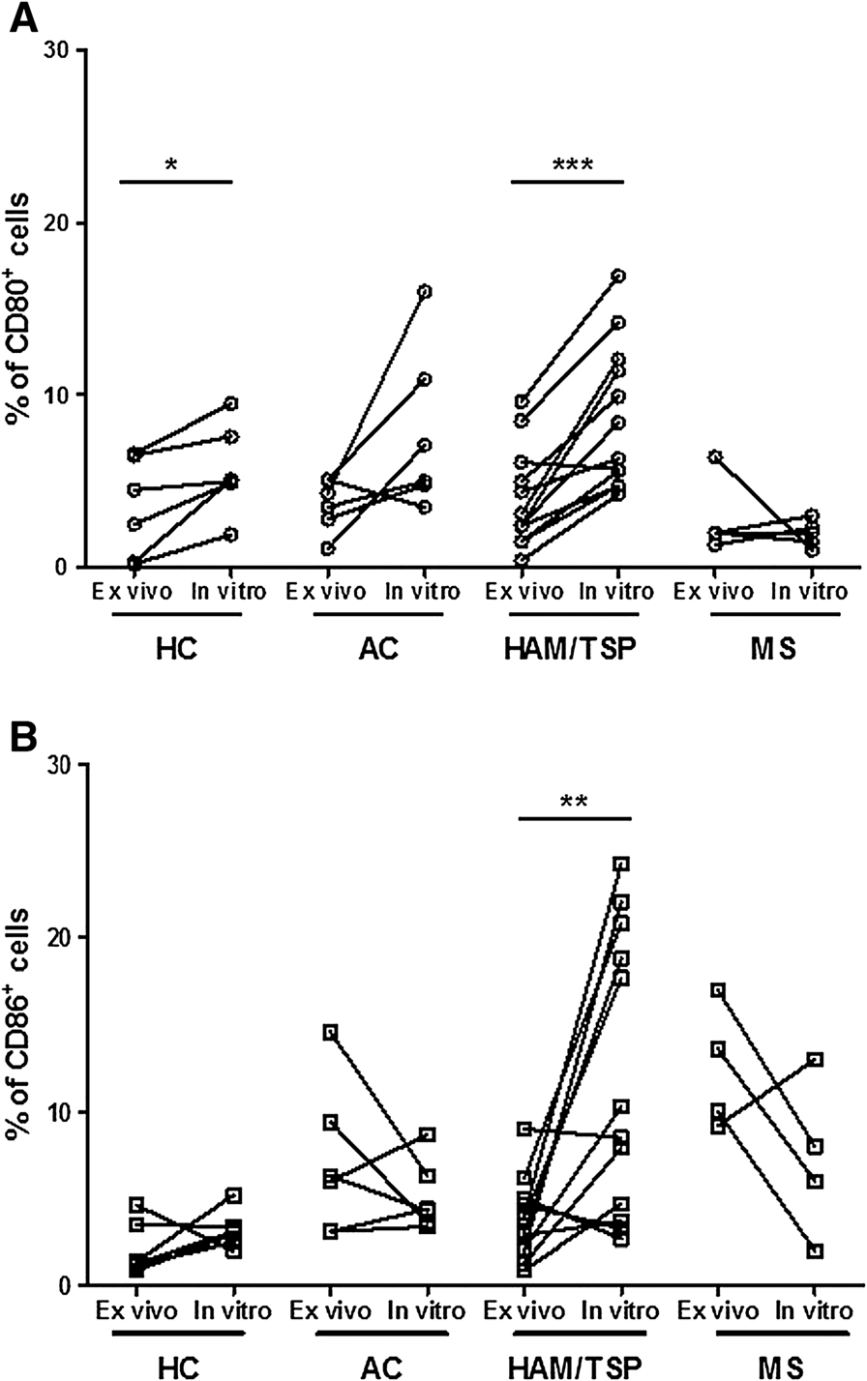
**Expansion of CD80**^**+**^**and CD86**^**+ **^**lymphocytes upon *in vitro *culture of PBMCs of HAM/TSP patients but not in HTLV-1 carriers and MS patients.** CD80 and CD86 expression in PBMCs was quantified before (*ex vivo*) and after 48 h of *in vitro* culture by flow cytometry. **(A)** Significant *in vitro* expansion of CD80^+^ cells in HAM/TSP patients (***p = 0.0001, *n* = 12) and HCs (*p = 0.049, *n* = 6). **(B)** Significant *in vitro* expansion of CD86^+^ cells in HAM/TSP patients only (**p = 0.0054; paired *t* test).

**Figure 7:**
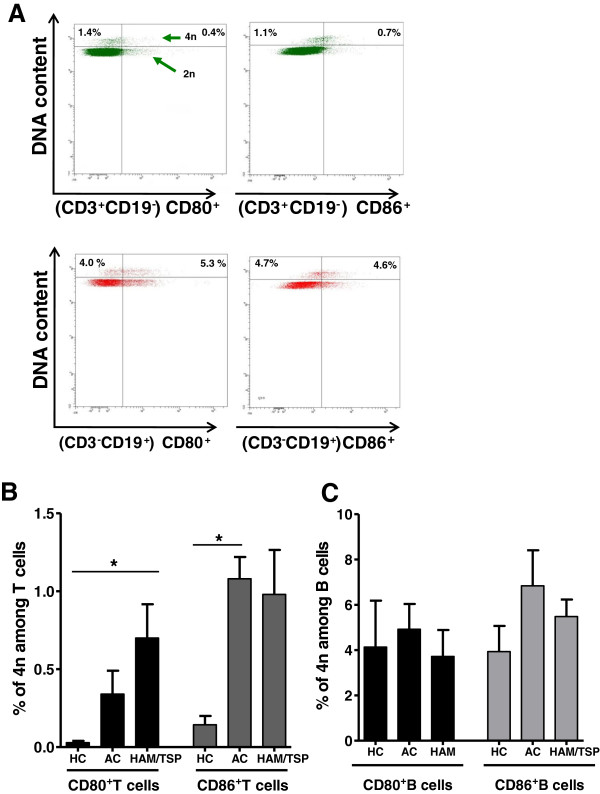
**Increased proliferation of CD80**^**+ **^**and CD86**^**+ **^**T cell subsets but not B cell subsets between HCs, ACs and HAM/TSP patients. (A)** PBMCs were cultivated *in vitro* for 48 h, and anti-CD3, anti-CD19, anti-CD80, anti-CD86 mAbs and Hoechst 33342 (DNA dye) staining was quantified using flow cytometry. Tetraploid (4n, proliferating) cells were gated on CD80- or CD86-expressing subsets of T (CD3^+^CD19^-^) cells or B (CD3^-^CD19^+^) cells. **(B,C)** Proliferating CD80^+^ T cells were significantly increased in HAM/TSP vs. HCs only, and proliferating CD86^+^ T cells were significantly increased in ACs vs. HCs only **(B)**, whereas there was no significant difference in proliferation in any B cell subset **(C)**. (*n* = 3 for HCs and *n* = 5 for ACs and HAM/TSP patients, Kruskal-Wallis with Dunn’s multiple comparison post test, *p < 0.05).

As shown in Figure 6A and B, in HAM/TSP patients, at 48 h of *in vitro* culture, we observed a significant (2.2-fold) increase in CD80^+^ lymphocytes and a significant (3.2-fold) increase in CD86^+^ lymphocytes versus *ex vivo* levels. In contrast, only CD80, but not CD86, expression in HCs (and neither CD80 nor CD86 expression in ACs) was increased upon *in vitro* culture. However, the ratio of CD86:CD80 in HCs, ACs and HAM/TSP patients did not alter significantly upon *in vitro* culture (data not shown). In both HCs and HAM/TSP patients, B cells were the main *in vitro* reservoir of CD80^+^ and CD86^+^ lymphocytes (data not shown), in agreement with previous results observed by Genç et al. in MS patients and HCs [33]. Since we observed an *in vitro* expansion in CD80^+^ and CD86^+^ lymphocytes in HAM/TSP, we investigated whether a similar phenomenon occurred in MS. However, there was no significant expansion in either the percentage or MFI of CD80 or CD86 in either B cells or total lymphocytes upon *in vitro* culture of PBMCs of MS patients (Figure 6A and B). As such, *in vitro* expansion of these subpopulations is not common to neuroinflammatory diseases but rather a HAM/TSP-specific phenomenon. Regarding the role of the virus, the *in vitro* increase in CD80^+^ T cells (but not CD86^+^ T or CD80^+^/CD86^+^ B cells) was significantly correlated to the proviral load in both HTLV-1 infected individuals (*p* = 0.013, Spearman’s *r* = 0.66, *n* = 13) and HAM/TSP patients (*p* = 0.0046, Spearman’s *r* = 0.91, *n* = 8). In contrast, spontaneous lymphoproliferation in HAM/TSP patients, as measured by [^3^H]-thymidine incorporation, was positively correlated to all four lymphocyte subsets, i.e., CD80^+^ and CD86^+^ T and B lymphocytes (data not shown). Therefore, we analyzed specific T and B cell subsets for short-term (48-h) spontaneous proliferation using a sensitive flow cytometric assay. This short-term assay allowed simultaneous, single-cell analysis of CD80, CD86 and proliferation in both B and T cells (Figure 7A) and also eliminated the possible bias of selective B or T cell apoptosis during long-term (5-day) *in vitro* culture using [^3^H]-thymidine incorporation. As shown in Figure 7B, proliferation of the CD80^+^ T cell subset in HAM/TSP patients, but not in ACs, was significantly higher than in HCs. On the other hand, proliferation of the CD86^+^ T cell subset in ACs, but not in patients, was significantly higher than in HCs. This once more suggests a differential, i.e., deleterious vs. protective, regulation of both costimulatory molecules. We found no significant difference in proliferation of CD80^+^ or CD86^+^ B cell subsets between HCs, ACs and HAM/TSP patients (Figure 7C). Taken together, these results imply that increased *ex vivo* T cell CD80 and CD86 expression (Figure 2A) reflects increased proliferation of CD80^+^/CD86^+^ T cell subsets, whereas increased *ex vivo* B cell CD80 expression in both HTLV-1 infected individuals and HAM/TSP patients (Figure 2B) is probably not mediated by increased proliferation of the CD80^+^ B cell subset.

### IFN-β preferentially stimulates B cell CD86 expression in HCs and HAM/TSP patients

**Figure 8:**
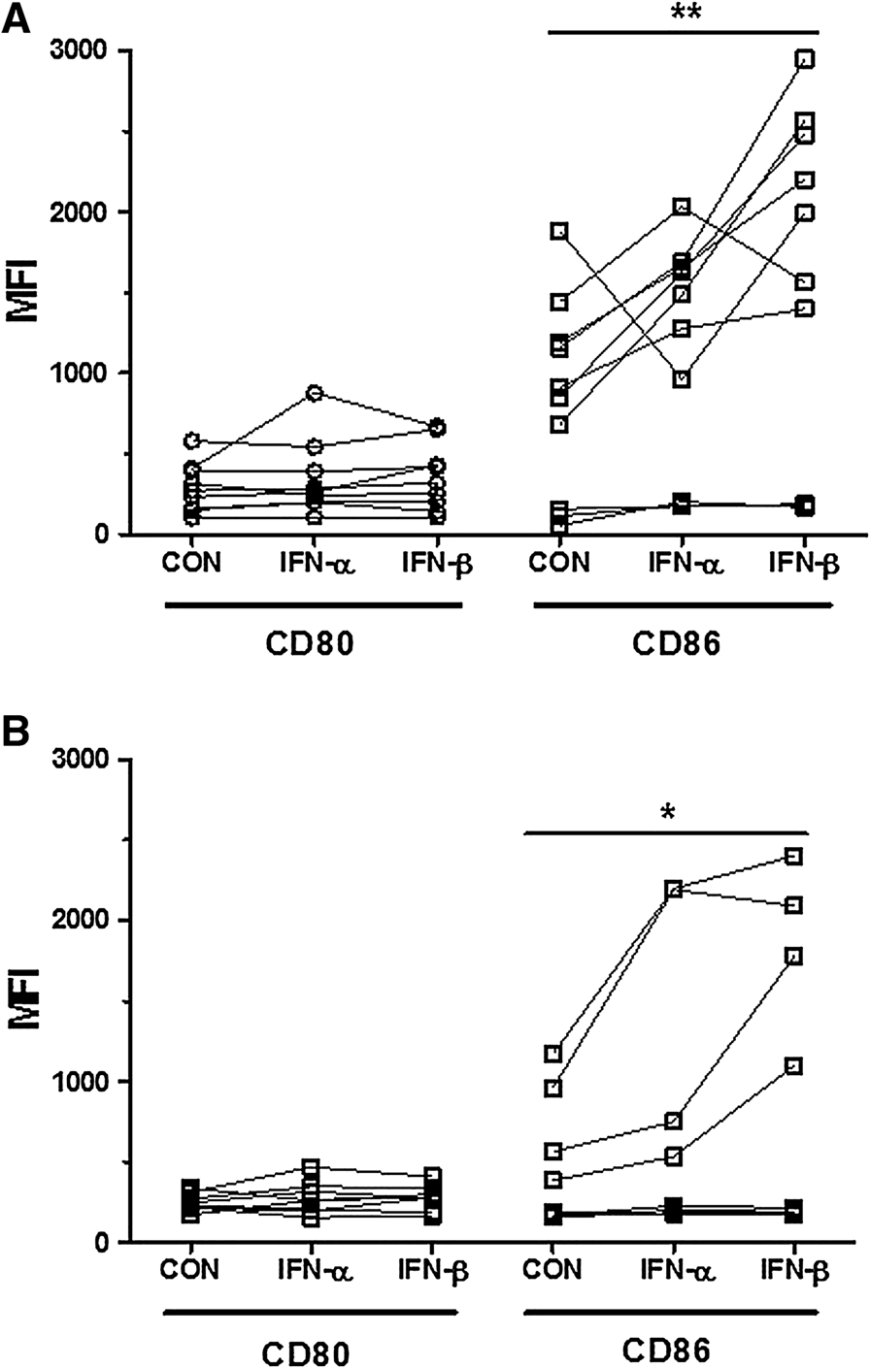
**Expression of CD80 and CD86 as MFI in B cells of HAM/TSP patients and HCs following *in vitro *treatment with IFN-α and IFN-β. (A,B)** IFN-β but not IFN-α enhanced expression of CD86 in B cells in both **(A)** HCs (**p = 0.0070, *n* = 10) and **(B)** HAM/TSP patients, (*p = 0.023, *n* = 8). (*p < 0.05, **p < 0.01, ***p < 0.001; ANOVA with Bonferroni’s multiple comparison post test).

In both HCs and HAM/TSP patients, percentages of CD80^+^ or CD86^+^ total lymphocytes as well as T and B cells did not increase significantly on treatment with IFN-α or IFN-β (data not shown), whereas the increase in MFI was more pronounced. As shown in Figure 8A and B, stimulation with IFN-α or IFN-β was unable to significantly modulate CD80^+^ B lymphocyte expression (MFI) in HCs as well as in HAM/TSP patients. On the other hand, IFN-β but not IFN-α significantly enhanced CD86 B lymphocyte expression (MFI) in both HCs and HAM/TSP patients. Upon IFN-β stimulation, the induction of CD86 expression (MFI) was comparable in both groups, namely HCs (1.9 fold) and HAM/TSP patients (2.2 fold). Similarly, in both groups the HCs and HAM/TSP patients, there was a significant increase in the ratio of CD86:CDs80 in B lymphocytes with IFN-β (*p* = 0.011 and *p* = 0.019, respectively) with a similar fold increase (1.6 fold and 1.7 fold, respectively). No significant increase was observed in the ratio for IFN-α in either HCs or HAM/TSP patients (*p* > 0.20 for both, data not shown).

To investigate the *in vivo* relevance of our *in vitro* data, we compared the *in vivo* and *in vitro* effect of IFN-β in MS patients. In keeping with Genc et al. [33,49], after 1 month of *in vivo* treatment with IFN-β, we observed a 1.72-fold decrease in the percentage of CD80^+^ B lymphocytes (*p* = 0.089) and a 1.5-fold increase in the percentage of CD86^+^ B lymphocytes (*p* < 0.0001) but not in total lymphocytes. In addition, upon *in vitro* treatment with IFN-β, the percentage of CD80^+^ B lymphocytes was downregulated in five of six MS patients (*p* < 0.0001), while the CD86^+^ B lymphocyte level was highly variable (data not shown), similar to the observations of Ramgolam et al. [50]. Thus, both *in vivo* and *in vitro* IFN-β treatment can decrease CD19^+^CD80^+^ and increase CD19^+^CD86^+^ B cells in MS patients, but with a strikingly high inter-patient variability consistent with previous studies [33,50]. Given this strong inter-patient variability, we recalculated IFN stimulation as % of control baseline values (MFI IFN-stimulated:MFI unstimulated cells) to enable a direct comparison between patient groups. We observed a highly similar *in vitro* response (mean % increase ± SEM) to IFN-β for CD80, CD86 and another IFN-regulated (Rep et al., 1999; Van Weyenbergh et al., 2001) surface molecule (Fas/CD95, data not shown) in both HAM/TSP and MS patients. This reveals that, despite immune dysregulation in patients with neuroinflammatory disease, the immunomodulatory potential of IFN-β is unaltered.

## Discussion

Until now, there have been no validated biomarkers for clinical monitoring of HAM/TSP patients for either disease progression or therapeutic response to any of the drugs currently used. The biomarkers proposed thus far in HAM/TSP either have conflicting results (as observed in proviral load and viral tax mRNA) or remain to be validated in other cohorts, namely viral HBZ mRNA [51] and host CD4^+^CD25^+^CCR4^+^Foxp3^-^IFN-γ^+^ T cells [52]. We hypothesized that *ex vivo* expression of CD80 and CD86 as well as the *in vitro* effects of IFN-α and IFN-β on their expression could reveal biomarkers for possible clinical use in HAM/TSP. In this study, which is the first to recruit HAM/TSP patients from two endemic countries (Brazil and Peru), we reveal CD80^+^ B cells as a novel host biomarker for disease severity (Figure 3A), while highlighting a possible protective role for CD86^+^ B cells, which are preferentially upregulated by IFN-β in HAM/TSP, both uncorrelated to proviral load.

B cell-expressed CD80/CD86 has been shown to drive pathogenesis in autoimmunity [53-55]. Despite their largely overlapping functions in T cell activation and immune upregulation in autoimmunity [28], CD80 and CD86 on APCs also play additional individually distinct roles, which cannot be substituted for by the other [56,57]. In murine models of graft arterial disease [58] and sepsis [59], CD80 is associated with proinflammatory cytokine stimulation, while CD86 plays a protective role mediated through IL-4 [58] or IL-10 [60] production. Further, CD80 and CD86 are differentially regulated in different tissue compartments [54,61] as well as cell types ([33], this study). Given the increase in CD80^+^ B cells in the CSF of MS patients [62,63], migration of CD80^+^ B cells from peripheral blood into the CNS is an intriguing possibility in HAM/TSP. In MS patients, increased B cell CD80 expression corresponds to exacerbations [33], and pronounced expression of CD80 i has been demonstrated in early active plaques [64]. In addition, a recent genome-wide association study implicated CD80, but more strongly CD86, in MS susceptibility [65]. CD80 is thought to play a key role in persistent infections or chronic inflammatory conditions [66], while CD86 plays a dominant role in initiating immune reactions [67]. Although not demonstrated in HTLV-1 infected patients, a positive correlation between spontaneous lymphoproliferation and CD80 expression was observed in HTLV-2-infected patients [26], indicating a possible mechanism for simultaneous upregulation of both deleterious candidate biomarkers, IFN-γ [52] and CD80 [this study], in HAM/TSP pathogenesis. Among other cytokines, proinflammatory IFN-γ transcripts are upregulated in HAM/TSP patients and seropositive carriers when compared to HCs [68]. *In vitro* studies in PBMCs of HAM/TSP patients and HTLV-1 carriers have indicated that drugs targeting TNF-α resulted in concomitant lowering of IFN-γ [69], implying that a common mechanism, at least in part, regulates the two cytokines. As discussed by Moens et al., miR-155 could serve as a candidate molecule to modulate IFN-γ production in HAM/TSP patients in parallel to its pathogenic role in MS and EAE [70]. In MS, endogenous IFN-γ significantly correlates to disability [71], and exogenous IFN-γ causes disease exacerbation [72]. Blocking B7-mediated activation causes long-term inhibition in EAE [73]. More specifically, blocking of CD80 inhibits EAE, while blocking of CD86 may even aggravate disease [29,55]. Finally, only CD80 interacts with PD-L1, which is required for the maintenance of peripheral T cell tolerance [74] and participates in EAE pathogenesis [75]. Taken together, in both autoimmune and infectious diseases a substantial amount of data is available on the differential regulation of CD80 vs. CD86, supporting our hypothesis of deleterious and protective roles for CD80^+^ and CD86^+^ B cells, respectively, in HAM/TSP pathogenesis.

Two possible mechanisms might be responsible for the observed differential expression of CD80/CD86 in B cells, either de novo synthesis induced by host transcription factors and/or viral transactivator tax and HBZ or a selective proliferation of CD80^+^ vs. CD86^+^ B cells. Some of the principal players among host transcriptional factors are interferon regulatory factor (IRF)-1, nuclear factor (NF)-κB, and signal transducer and activator of transcription (STAT)-1. IRF-1 [32] as well as NF-κB [76] exert transcriptional control in upregulating CD80. Increased proinflammatory IFN-γ is capable of inducing IRF-1 directly or via STAT-1 [77]. Further, IFN-γ is capable of a five-fold induction of STAT-1 and IRF-1 in B cells [78], while stimuli such as TNF activating the NF-κB pathway [79,80] could account for CD80 upregulation. On the other hand, the CD86 gene promoter contains two STAT-1 binding sites, which might explain its upregulation by IFNs [81]. We therefore hypothesize a relationship between the two host cell biomarkers associated with HAM/TSP disease severity: CD4^+^CD25^+^CCR4^+^Foxp3^-^IFN-γ^+^[52] and CD19^+^CD80^+^ cells (this study). It is plausible that CD4^+^CD25^+^CCR4^+^Foxp3^-^IFN-γ^+^ cells could influence the upregulation of CD80 in B cells through IFN-γ stimulation. In MS, it has been shown that endogenous IFN-γ significantly correlates to disability [71] and exogenous IFN-γ causes disease exacerbation [82].

**Figure 9:**
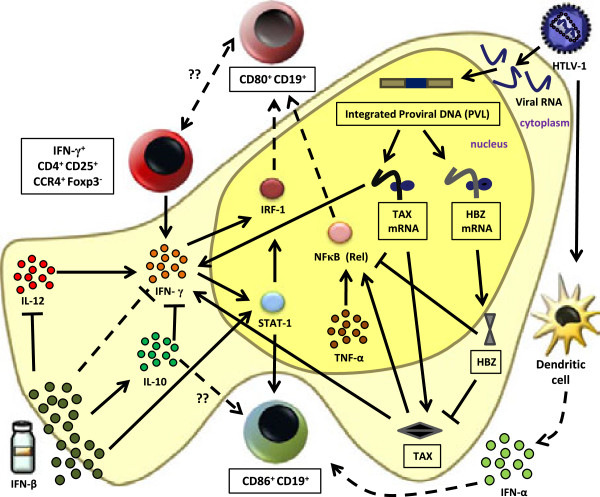
**Proposed model integrating viral and host biomarkers for disease severity and the effect of IFN-β in HAM/TSP.** Viral tax could be driving the upregulation of CD80-expressing B cells in an IFN-γ/IRF-1 [77,78] or TNF/NF-κB [79,80,84] dependent manner. HBZ downregulates tax [103] and the NF-κB pathway [104], thereby possibly downregulating CD80 expression. IFN-γ-producing CD4^+^CD25^+^CCR4^+^Foxp3^-^ host cells [52] could positively influence CD80 expression in a STAT-1/IRF-1-dependent manner. IFN-β induced downregulation of CD80 expression in B cells [33] could be, in part, due to its antagonistic effect on IFN-γ [100-102] as well as modulation of cytokines (IL-10 and IL-12) [105,106] regulating IFN-γ. CD86 expression by IFN-β may be driven via STAT-1 or possibly via IL-10. HTLV-1-infected pDCs produce endogenous IFN-α [91], which might upregulate B cell CD86 expression *in vivo*. The continuous arrows and inhibition lines indicate published data, while the discontinuous lines indicate hypothetical links drawn from this study. The proinflammatory molecules/cells are in *shades of brown/red*, while anti-inflammatory molecules/cells are in *shades of green*. Proposed biomarkers for HAM/TSP are enclosed in *boxes*.

As shown in Figure 9, we propose a scheme integrating the proposed viral and host biomarkers for HAM/TSP disease severity that might help explain differential CD80 and CD86 expression. The regulation and interplay between these host factors could be driven by viral tax or HBZ. Although data on the role of HBZ in HAM/TSP have been scarce, Saito et al. recently demonstrated a positive correlation between HBZ mRNA and disease severity [51]. This increase in the HBZ mRNA level could likely entail an increase in HBZ protein, either of which may be involved in HAM/TSP pathogenesis based upon their differential role in ATL [83]. On the other hand, tax is thought to be responsible for NF-κB activation [84]. Tax contributes greatly to inflammatory reactions related to the CNS because of the overexpression of TNF-α and IFN-γ [85-87]. The increase in CD80^+^ B cells could thus be driven by tax in an IFN-γ/IRF-1- or TNF-α/NF-κB-dependent manner. Therefore, it would be interesting to determine whether CD4^+^CD25^+^CCR4^+^Foxp3^-^IFN-γ^+^ cells could be (a subset of) the T cells expressing CD80 or CD86, given their role in EAE [88]. We found that *in vitro* proliferation of HAM/TSP PBMCs positively correlates to both CD80^+^CD3^+^ and CD86^+^CD3^+^ cells (Van Weyenbergh et al., unpublished data). However, since neither CD80^+^ nor CD86^+^ T cells correlated to disease severity, the CD4^+^CD25^+^CCR4^+^Foxp3^-^IFN-γ^+^ cells are probably a distinct T cell population. Given that B cells proliferate to a lesser extent than T and NK cells [47], the observed increase in CD80/CD86 expression in B cells might be due to de novo expression rather than B cell proliferation, as suggested by Figure 7C. Several mechanisms might account for the observed increase in *ex vivo* and/or *in vitro* expression of CD80/CD86. For instance, (1) induction of anti-inflammatory type I IFN, which upregulates CD80/CD86 [32,33,89], (2) elevation of proinflammatory IFN-γ levels [17,90] or (3) a delicate cytokine balance regulated by the two types of IFNs driving the expansion of CD80^+^/CD86^+^ lymphocytes. HTLV-1 virions infecting pDC are capable of inducing IFN-α production [91]. Accurate quantification of type I IFN levels *ex vivo* or *in vitro* is difficult, given that they are present close to or below detectable limits. Although an IFN-inducible signature was recently identified in HAM/TSP [92], the same report showed no apparent differences in endogenous type I IFN production between HCs, ACs and HAM/TSP patients [92]. However, this predominantly myeloid gene signature identified in whole blood samples did not include CD80 or CD86, which might indicate a cell type-specific regulation of these molecules. Hence, we investigated the effect of IFN-α/β *in vitro* stimulation in lymphocyte subsets in HCs and HAM/TSP patients. This allowed for a direct, quantitative and qualitative comparison between both cytokines (IFN-α and IFN-β), which has not been previously carried out in HAM/TSP. *In vivo* treatment with IFN-α has shown clinical benefits in HAM/TSP patients [10,14,15,39,40]. IFN-α studies have mainly focused on T cell subsets and cytokines, indicating a decrease in the ratio of CD4:CD8 cells, particularly the CD4^+^CD25^+^ and CD4^+^CD45RO^+^ T subsets [93]. Few studies have addressed the biological and immunoregulatory properties of IFN-β in the context of HTLV-1 infection. Nonetheless, the pleiotropic effects of IFN-β are thought to play a protective role in host mononuclear cells upon *in vitro* infection [94,95]. *In vivo*, in HAM/TSP, the only IFN-β1a trial thus far resulted in amelioration of motor functions with a corresponding decrease in spontaneous lymphoproliferation, tax mRNA as well as HTLV-1-specific CD8^+^ cells [15]. However, to date, no studies have investigated the effect of IFN-α or IFN-β on B cells in HAM/TSP patients. In contrast, data on the mechanisms of action of IFN-β *in vivo* are abundant in MS [96-98], with IFN-β therapy effectively upregulating CD86 in monocytes and B cells [33,34,89,99] while downregulating CD80 *in vivo*[49] but not *in vitro*[33]. This discrepancy might be due to an IFN-γ-mediated mechanism, since IFN-γ aggravated disease [72], while IFN-β resulted in clinical benefit [37], and reduced serum IFN-γ levels [100]. *In vitro*, IFN-β not only downregulates IFN-γ [100,101], but also antagonizes the effect of IFN-γ upon CD64 [102] and MHC class II expression [29].

Despite IFN-α and IFN-β engaging the same receptors, differential outcomes of stimulation can be attributed to their different affinities and rate constants of interaction with IFN type I receptor subunits, IFN-α receptor (IFNAR) 1 and IFNAR2 [107,108], and the differential use of the beta subunit of IFNAR1 [109]. Recently, de Weerd et al. provided structural evidence of the specific binding of IFN-β to IFNAR1 in an IFNAR2-independent manner [110]. Further, IFN-α and IFN-β lead to differential downregulation and routing of IFNAR2 [107]. The tyrosine kinases required for IFNα/β-mediated STAT-1 activation, tyrosine kinase (Tyk) 2 and Janus kinase (Jak) 1, are associated with their substrate type I interferon receptor chains, IFNAR1 and IFNAR2, respectively [111]. Genetic deletion of Jak1 results in the inability to respond to IFN-α or IFN-β [112]. In contrast, deletion of Tyk2 causes a complete lack of IFN-α responsiveness [113], whereas IFN-β can still elicit a limited signaling response in the absence of Tyk2 [114]. Interestingly, Tyk2 is also an MS susceptibility gene [115]. Selective IFN-β-induced activation of Jak1 and higher STAT-1 phosphorylation upon IFN-β vs. IFN-α treatment were observed [116] in both human myocardial fibroblasts and vascular endothelial cells [117], and a superior antiproliferative effect of IFN-β over IFN-α was shown in Ewing’s sarcoma cells *in vitro*[118]. Preferential induction of CD86 over CD80 *in vivo* has been demonstrated in a mouse model for influenza infection, in which type I IFN-mediated signals were responsible for upregulation of CD86 in B cells [119]. In addition, CD86 upregulation in human immunodeficiency virus as well as simian immunodeficiency virus co-infection of dendritic cells *in vitro* was IFN-β but not IFN-α-dependent [120]. IFN-β-deficient or interferon type I receptor-deficient (IFNAR^−/−^) mice developed more severe EAE, reinforcing a protective role for type I IFN [121,122].

Similar to our results, a recently identified CD11b^+^CD11c^+^B220^+^CD21^-^ B cell population was predominant in female aged and autoimmune disease-prone mice in response to TLR-7 signaling [123]. Interestingly, regulatory B cells secreting IL-10 have been shown to play a protective role in EAE [124] and MS [125], but the possible relationship between regulatory B cells and CD80 vs. CD86 B cell expression is still undefined.

Finally, our results encourage future clinical trials with IFN-β in HAM/TSP and open up avenues to B cell- or CD80-directed therapies in HAM/TSP. Low numbers of circulating B cells and a corresponding increase in the T:B ratio have been previously reported in HAM/TSP patients [126]. Furukawa et al. observed an *ex vivo* increase in phosphatidylserine exposure in B cells of HAM/TSP patients, which was reversible upon *in vitro* culture [48]. To date, these have been the only investigations with regard to B cells in HAM/TSP. However, B cell depletion has provided clinical benefit in rheumatoid arthritis as well as MS [111-115]. Anti-CD20 therapy depleted activated B cells in PBMCs and CSF, downregulated proinflammatory cytokine responses of CD4^+^ and CD8^+^ cells [127,128], reduced inflammatory brain lesions [129,130], and lowered CSF T and B cell levels. In addition to anti-CD20 antibodies, anti-CD80 antibodies might represent a novel therapeutic option in both HAM/TSP and MS. Anti-CD80 clinical trials have demonstrated clinical benefit with no severe adverse effects in both psoriasis [131] and CD80^+^ B cell lymphoma [132], providing proof of concept for *in vivo* targeting of CD80 in human disorders.

## Conclusion

We propose two novel biomarkers for future clinical use in HAM/TSP: CD80^+^ B cells positively correlating to disease severity and CD86^+^ B cells preferentially induced by IFN-β. Our results reveal B cellimmunotherapy (with proven clinical benefit in MS) to be a plausible therapeutic alternative in HAM/TSP and also suggest CD80-directed immunotherapy, unprecedented in both HAM/TSP and MS.

## Competing interest

The authors declare that they have no competing interests.

## Authors’ contributions

SMM carried out most experiments, analyzed data and wrote the manuscript. DD carried out initial HAM/TSP *in vitro* experiments. DB provided MS patient samples. RKh carried out flow cytometry experiments. SVS carried out initial *ex vivo* experiments and collected HAM/TSP clinical data. RKr diagnosed and followed Brazilian HAM/TSP patients. GL quantified the proviral load and assisted with *in vitro* experiments. CA provided Peruvian HAM/TSP patient samples. MT coordinated experimental work in Peru. EG coordinated the Peruvian patient cohort. AMV participated in the study design and helped to draft the manuscript. BGC coordinated the Brazilian patient cohort. RL diagnosed and followed MS patients and designed the IFN-β/MS study. JVW conceived the study, designed and performed experiments, analyzed data and helped to draft the manuscript. All authors read and approved the final manuscript.

## Supplementary Material

Additional file 1: Figure S1 Correlation between CD86:CD80 ratio in B cells and disease severity. Ratio of the *ex vivo* levels of CD86 and CD80 in B cells negatively correlated to Kurtzke’s EDSS. (*p = 0.036, Pearson’s *r* = − 0.50, both the ratio CD86:80 and EDSS are normally distributed, *n* = 18). Click here for file

Additional file 2: Figure S2 CD80 levels discriminate patients with impaired mobility in HAM/TSP and MS. (A) *Ex vivo* CD80^+^:CD19^+^ ratio greater than 0.23 (*green filled circle*, A) (ROC curve, **p = 0.0010, AUC = 0.96) differentiate HAM/TSP patients with EDSS greater >4 (*n* = 10) from those with EDSS ≤4 (*n* = 8). (B) *Ex vivo* levels of CD19^+^CD80^+^ cells greater than 8.4% (*green filled circle*, B) (ROC curve, *p = 0.023, AUC = 0.85) differentiate patients with active MS (*n* = 6) from patients with non-active MS (*n* = 10). Click here for file
