# Supplementary material for: CD80+ and CD86+ B cells as biomarkers and possible therapeutic targets in HTLV-1 associated myelopathy/tropical spastic paraparesis and multiple sclerosis
Source: J Neuroinflammation. 2014 Jan 29;11:18. doi: 10.1186/1742-2094-11-18 (PMC3922160; doi:10.1186/1742-2094-11-18)
Supplement: Additional file 1: Figure S1 — Correlation between CD86:CD80 ratio in B cells and disease severity. Ratio of the ex vivo levels of CD86 and CD80 in B cells negatively correlated to Kurtzke’s EDSS. (*p = 0.036, Pearson’s r = − 0.50, both the ratio CD86:80 and EDSS are normally distributed, n = 18). [file 1742-2094-11-18-S1.doc]

SUPPLEMENTARY FIGURES:

Additional file 1: Figure S1.

Additional file 1: Figure S1. Correlation between CD86/CD80 ratio in B cells and disease severity. **Ratio of the *ex vivo* levels of CD86 and CD80 in B cells negatively correlated to Kurtzke’s EDSS. (*p=0.036, Pearson’s r= - 0.50, both ratio CD86/80 and EDSS are normally distributed, n=18)**
