# Supplementary material for: CD80+ and CD86+ B cells as biomarkers and possible therapeutic targets in HTLV-1 associated myelopathy/tropical spastic paraparesis and multiple sclerosis
Source: J Neuroinflammation. 2014 Jan 29;11:18. doi: 10.1186/1742-2094-11-18 (PMC3922160; doi:10.1186/1742-2094-11-18)
Supplement: Additional file 2: Figure S2 — CD80 levels discriminate patients with impaired mobility in HAM/TSP and MS. (A) Ex vivo CD80+:CD19+ ratio greater than 0.23 (green filled circle, A) (ROC curve, **p = 0.0010, AUC = 0.96) differentiate HAM/TSP patients with EDSS greater >4 (n = 10) from those with EDSS ≤4 (n = 8). (B) Ex vivo levels of CD19+CD80+ cells greater than 8.4% (green filled circle, B) (ROC curve, *p = 0.023, AUC = 0.85) differentiate patients with active MS (n = 6) from patients with non-active MS (n = 10). [file 1742-2094-11-18-S2.doc]

Additional file 2: Figure S2.

A B

Additional file 2: Figure S2. CD80 levels discriminate patients with impaired mobility in HAM/TSP and MS. **(A) *Ex vivo* CD80^+^/CD19^+^ ratio greater than 0.23 (green filled circle, Fig. A) (ROC curve, **p=0.0010, AUC=0.96) differentiate HAM/TSP patients with EDSS greater >4 (n=10) from those with EDSS ≤4 (n=8). (B) *Ex vivo* levels of CD19^+^CD80^+^ cells greater than 8.4% (green filled circle, Fig. B) (ROC curve, *p=0.023, AUC=0.85) differentiate patients with active MS (n=6) from patients with non-active MS (n=10).**
